# FOXP3^+^ T Cells Recruited to Sites of Sterile Skeletal Muscle Injury Regulate the Fate of Satellite Cells and Guide Effective Tissue Regeneration

**DOI:** 10.1371/journal.pone.0128094

**Published:** 2015-06-03

**Authors:** Alessandra Castiglioni, Gianfranca Corna, Elena Rigamonti, Veronica Basso, Michela Vezzoli, Antonella Monno, Albert E. Almada, Anna Mondino, Amy J. Wagers, Angelo A. Manfredi, Patrizia Rovere-Querini

**Affiliations:** 1 Division of Regenerative Medicine, Stem Cells and Gene Therapy, San Raffaele Scientific Institute & Vita-Salute San Raffaele University, Milan, Italy; 2 Department of Stem Cell and Regenerative Biology, Harvard University, Cambridge, MA, United States of America; 3 Joslin Diabetes Center, Boston, MA, United States of America; 4 Howard Hughes Medical Institute, Cambridge, MA, United States of America; 5 Harvard Stem Cell Institute, Cambridge, MA, United States of America; 6 The Paul F. Glenn Laboratory for the Biological Mechanisms of Aging, Harvard Medical School, Boston, MA, United States of America; 7 Division of Immunology, Transplantation and Infectious Disease, San Raffaele Scientific Institute & Vita-Salute San Raffaele University, Milan, Italy; University of Louisville School of Medicine, UNITED STATES

## Abstract

Muscle injury induces a classical inflammatory response in which cells of the innate immune system rapidly invade the tissue. Macrophages are prominently involved in this response and required for proper healing, as they are known to be important for clearing cellular debris and supporting satellite cell differentiation. Here, we sought to assess the role of the adaptive immune system in muscle regeneration after acute damage. We show that T lymphocytes are transiently recruited into the muscle after damage and appear to exert a pro-myogenic effect on muscle repair. We observed a decrease in the cross-sectional area of regenerating myofibers after injury in Rag2^-/-^ γ-chain^-/-^ mice, as compared to WT controls, suggesting that T cell recruitment promotes muscle regeneration. Skeletal muscle infiltrating T lymphocytes were enriched in CD4^+^CD25^+^FOXP3^+^ cells. Direct exposure of muscle satellite cells to *in vitro* induced Treg cells effectively enhanced their expansion, and concurrently inhibited their myogenic differentiation. *In vivo*, the recruitment of Tregs to acutely injured muscle was limited to the time period of satellite expansion, with possibly important implications for situations in which inflammatory conditions persist, such as muscular dystrophies and inflammatory myopathies. We conclude that the adaptive immune system, in particular T regulatory cells, is critically involved in effective skeletal muscle regeneration. Thus, in addition to their well-established role as regulators of the immune/inflammatory response, T regulatory cells also regulate the activity of skeletal muscle precursor cells, and are instrumental for the proper regeneration of this tissue.

## Introduction

Immune cells are rare in the healthy skeletal muscle; however, they are swiftly recruited in response to injury, where their concentration can exceeds 100,000/mm^3^ of tissue [1]. Inflammation coincides with muscle repair and regeneration, and a role for the innate immune system in these latter events has been convincingly documented [2–5]. Comparatively less is known about the role of the adaptive immune system in these processes. Lymphocytes consistently infiltrate the muscle in conditions of chronic injury, including muscular dystrophies [6, 7]. Their function, whether protective or deleterious, is largely unknown. An acute injury of the tissue, such as the one elicited by the injection of cardiotoxin (CTX) into murine muscle, prompts the release of endogenous inflammatory signals by necrotic myofibers and activates a transient, self-limited leukocyte infiltration of the tissue, which culminates in complete regeneration due to the activation, proliferation and timely fusion of muscle progenitor cells [8, 9]. In this study, we investigated whether this response involves the activity of lymphocytes, and whether lymphocytes are required for the eventual healing of the tissue. Through flow cytometric characterization of the leukocytes that infiltrate the muscle after CTX injury, we show that a specific CD4^+^CD25^+^FOXP3^+^ population of T cells is present in the skeletal muscle during the phase of regeneration in which satellite cells are actively proliferating. Using gene-modified mice that specifically lack adaptive immune cells and through co-culture of isolated T and satellite cells, we show that T regulatory cells are involved in muscle healing and that they directly interact with satellite cells, inducing their expansion.

## Materials and Methods

### Mice

C57BL/6 mice from Charles River and Rag2^-/-^ γ-chain^-/-^ mice from Taconic were housed in the San Raffaele Institute and in the Joslin Diabetes Center Facilities. Pax7-Zsgreen mice (kindly provided by Dr. Michael Kyba, University of Minnesota) and FOXP3-GFP mice (kindly provided by Dr. Maria-Grazia Roncarolo, San Raffaele Institute, Milano) were bred in the San Raffele Institute Facility. All the mice used for the experiments described in this paper were 8–10 week old females. Animal care and experimental protocols were approved by the Institutional Animal Care (IACUC) of the Joslin Diabetes Center and of the San Raffaele Institute.

### Muscle injury

*Tibialis anterior* and *Quadriceps* of anesthetized mice were injected once with CTX, *Naja mossambica mossambica* (Sigma Aldrich, 50 or 100 μL, 10 μM in saline). Mice were sacrificed and muscles retrieved 1, 3, 5, 7, 10, 15 and 20 days after. Injured muscles were collected and frozen or digested depending on the experiment.

### Immune infiltrate analysis

Single cells were obtained by enzymatic digestion of muscles with collagenase type IV (0.5 mg/ml, Sigma Aldrich) and dispase (3.5 mg/ml, Invitrogen) at 37°C for 40 min. Approximately 1-5X10^5^ cells were Fc blocked with rat anti-mouse CD16/CD32 (Mouse BD Fc Block, clone 2.4G2, 1:50) in PBS containing LIVE/DEAD Fixable Aqua Dead Cell Stain Kit (1:500, Invitrogen) for 30 min on ice. 30 min incubation was performed in PBS containing 5% FCS and 0.1mM EDTA using appropriate combinations of the antibodies. FITC: CD25 (BD, clone 7D4, 1:100), Ly6G (Biolegend, 1A8, 1:200). PE: CD8 (BD, clone 53–6.7, 1:50), CD19 (BD, clone 1D3, 1:200), CD210 (IL10RA, Biolegend, clone 1B1.3a, 1:20). PERCP: CD4 (BD, clone RM4-5, 1:100), NK1.1 (BD, clone PK136, 1:100). PERCP-Cy5.5: CD4 (Biolegend, clone RM4-5, 1:100). APC: CD11b (Biolegend, M1/70, 1:125) CD44 (BD, clone IM7, 1:200). PE-Cy7: CD3 (BD, clone 145-2C11, 1:65). APC-Cy7: CD45 BD, clone 30-F11, 1:125), CD69 (BD, clone H1.2F3, 1:100). V450: CD45 (BD, clone 30-F11, 1:125). Intracellular staining of FOXP3 (eBioscience, clone FJK-16s, 1:20) was performed using the Foxp3/Transcription Factor Staining Buffer Set (eBioscience) following manufacturer’s instruction. The cells were analyzed by flow cytometry (LSR Fortessa or LSRII, Diva Software, BD Bioscience and FlowJo, Tree Star, Inc).

### Satellite cells quantification

Injured and uninjured TA muscles from C57BL/6 mice were harvested at day 3 and 5 after CTX injection. Muscles were weighed and mononuclear cells were obtained by enzymatic digestion with 0.2% dispase and 0.05% collagenase II in DMEM (Invitrogen) at 37°C for 15 min. The cells were counted and the antibody staining was performed 30 min on ice in HBSS (Invitrogen) 2% DBS using appropriate combinations of the antibodies. APC Cy7: CD45 (BD, clone 30-F11, 1:200), CD11b (BD, clone M1/70, 1:200), TER119 (Biolegend, clone TER-119, 1:200). CXCR4 biotinilated (BD, clone 2B11/CXCR4, 1:100) followed by PE-Cy7 streptavidin (eBioscience, 1:200). APC conjugated Sca-1 (eBioscience, clone D7, 1:200). PE conjugated β1 integrin (BD, clone M1/69, 1:200) or purified β1 integrin (BD, clone M1/69, 1:100) followed by FITC conjugated goat anti-hamster IgG (eBioscience, 1:200) when PE conjugated CD210 (IL10RA, Biolegend, clone 1B1.3a, 1:20) antibody was used. Calcein Blue (Invitrogen) and PI were used to distinguish live cells.

### Morphometric analysis

C57BL/6 and Rag2^-/-^ γ-chain^-/-^ mice TA muscles were harvested, frozen and sectioned at 7 μm. Sections were fixed in 4% PFA for 10 minutes at room temperature. After 2 washes with in PBS, the tissue was incubated 1.5 hours at room temperature in 4% BSA, 5% FCS, 1% Triton-X in PBS. Tissue was stained with primary antibody (Abcam, chicken anti mouse laminin, 1:500) at 4°C overnight and with a secondary antibody (Invitrogen, anti-chicken Alexa Fluor 555, 1:500) 1 hour at room temperature. Specimens were counterstained with Hoechst 33342 (Molecular Probes) and analyzed using a Nikon Eclipse 55i microscope (Nikon). Images were captured with Digital Sight DS-5 M digital camera (Nikon) using Lucia G software (Laboratory Imaging). Cross-sectional areas of the myofibers were were quantified using ImageJ software.

### Quantitative real-time PCR analysis

Quantitative real-time PCR was performed on total muscle lysate or on CD3^+^ cells isolated from damaged muscles. Samples were homogenized and total cellular RNA was extracted from muscle using TRIZOL reagent (Applied Byosistems) or the RNeasy Micro Kit (Qiagen) following the manufacturer’s recommendations. RNA (1μg) was used for quantitative PCR (qPCR) analysis for first-strand synthesis of complementary DNAs (cDNAs) with the high-capacity cDNA Reverse Transcription kit (Applied Byosistems). qRT-PCR was performed using SYBR-green PCR Master Mix (Applied Byosistems). The level of each RNA was normalized to the corresponding level of GAPDH or Bactin messenger RNA (mRNA). The following primers were used: IL-10 (5’-ATTTGAATTCCCTGGGTGAGAAG-3’ forward; 5’-CACAGGGGAGAAATCGATGACA-3’ reverse), TGFβ (5’-CCCCACTGATACGCCTGAGT-3’ forward; 5’-AGCCCTGTATTCCGTCTCCTT-3’ reverse), IL27 (5’-CTGTTGCTGCTACCCTTGCTT-3’ forward; 5’-CACTCCTGGCAATCGAGATTC-3’ reverse), IL2 (5’-GTGCTCCTTGTCAACAGCG-3’ forward; 5’-GGGGAGTTTCAGGTTCCTGTA-3’ reverse) IFNγ (5’-CATTGAAAGCCTAGAAAGTCTG-3’ forward; 5’-CTCATGAATGCATCCTTTTTCG-3’ reverse), TNFα (5’-TCCCAGGTTCTCTTCAAGGGA-3’ forward; 5’-GGTGAGGAGCACGTAGTCGG-3’ reverse) CCR4 (5’-GGAAGGTATCAAGGCATTTGGG-3’ forward; 5’-GTACACGTCCGTCATGGACTT-3’ reverse), IL23 (5’-ATGCTGGATTGCAGAGCAGTA-3’ forward; 5’-ACGGGGCACATTATTTTTAGTCT-3’ reverse), IL17 (5’-TTTAACTCCCTTGGCGCAAAA-3’ forward; 5’-CTTTCCCTCCGATTGACAC-3’ reverse), IL6 (5’-TAGTCCTTCCTACCCCAATTTCC-3’ forward; 5’-TTGGTCCTTAGCCACTCCTTC-3’ reverse), IL4 (5’-GGTCTCAACCCCCAGCTAGT-3’ forward; 5’-GCCGATGATCTCTCTCAACTGAT-3’ reverse), Pax7 (5’-GACTCGGCTTCCTCCATCTC-3’ forward; 5’-AGTAGGCTTGTCCCGTTTCC-3’ reverse), MyoD (5’-ACGGCTCTCTCTGCTCCTTT-3’ forward; 5’-GTAGGGAAGTGTGCGTGCT-3’ reverse), IGF-1 (5′-GTGTGGACCGAGGGGCTTTTACTTC-3′ forward; 5′-GCTTCAGTGGGGCACAGTACATCTC-3′ reverse), GAPDH (5’-GCAAATTCAACGGCACAGTCAAG-3’ forward; 5’-GGTACAAACACTACCCACACTTG-3’ reverse), and β-actin (5’-TGCTGTCCCTGTATGCCTCT-3′ forward; 5’-GATGTCACGCACGATTTCC-3’ reverse).

### Western blot analysis

Single cells from injured muscles were obtained by enzymatic digestion of the tissue with collagenase type IV (Sigma Aldrich) (0.5 mg/ml) and dispase (Invitrogen) (3.5 mg/ml) at 37°C for 40 min. CD3^+^ cells were then isolated by magnetic sorting (CD3ε MicroBead Kit, Milteniy) following manufacturer’s instruction. Isolated CD3^+^ cells were lysed in RIPA buffer and protease inhibitors cocktail (Sigma Aldrich). Lysates were cleaned by centrifugation at 16,000 x g for 5 min at 4°C. For Western blot analysis, equal amounts of protein (20 μg) were resolved by SDS polyacrylamide gel electrophoresis and transferred onto Immobilon-P (Millipore). Antigens were detected using mouse anti-FOXP3 (1:500, clone 150D, Biolegend), rat anti-RORγT (1:250, clone B2D, eBioscience), mouse anti-GATA3 (1:200, clone HG3-31, Santa Cruz biotec), mouse anti-TBET (1:200, clone 4B10, Santa Cruz biotec), mouse monoclonal anti-β-actin (1:10000, clone AC15, Sigma Aldrich). All antibodies were diluted in TBST containing 5% non-fat milk. Incubation was performed 2 hours at room temperature for primary antibodies and 1 hour at room temperature for second step reagents. Primary antibodies were revealed with HRP-conjugated secondary antibodies (1:1000, GE Healthcare Europe GmbH) and a chemiluminescence kit (ECL, western blotting detection reagents, GE Healthcare Europe GmbH). When necessary, membranes were stripped with 0.1N NaOH for 10 min at room temperature.

### T cells and mouse satellite cells co-culture

CD4^+^ cells were isolated from lymph nodes of C57BL/6 mice by negative selection using anti-CD8 (clone KT1.5) and anti-MHC class II (clone M5/114) antibodies followed by sheep anti rat IgG Dynabeads (Invitrogen). Th0 and iTreg cells were cultured for 5 days in RPMI, 10% FCS, 50 μM βME (Invitrogen), 1% Pen-strep in plates previously coated with anti-CD3 and anti-CD28 antibodies (0,05–5 μg/ml). For iTreg induction TGFβ1 (2,5 μg/ml, R&D) and IL2 (20 ng/ml, R&D) were added to the medium. After 5 days, cells were harvested and co-cultured with satellite cells obtained from Pax7-ZsGreen mice. Muscles from Pax7-ZsGreen mice were dissected and dissociated by enzymatic digestion with collagenase type V (0.5 mg/ml, Sigma Aldrich) and dispase (3.5 mg/ml, Invitrogen) at 37°C for 40 min. On the same day naive T cells (CD4^+^) were isolated from lymph nodes and natural Treg (nTreg) were sorted from FOXP3-GFP mice (CD3^+^ GFP^+^ cells). FACS sorting was performed using the MoFLo system (Beckman Coulter). Satellite cells and T cells were co-cultured at a 1: 25-50-100-200 ratio in proliferating medium (F10, 20% horse serum, penicillin 100 U/ml, streptomycin 100 μg/ml, gentamycin 50 μg/ml, Gibco, with bFGF 5ng/ml, Sigma, added daily).

### BrdU proliferation assay

After 72 hours in proliferation medium, satellite cells were pulsed with BrdU (10 mM; Sigma-Aldrich) for 2 hours before fixation in 4% PFA and immunostaining for rat anti-BrdU antibody (final dilution: 1:100; R&D Systems). For anti-BrdU staining, cells were treated with HCl 2N, 30 min, 37°C and then with H_3_BO_3_ buffer 0.1M pH 8.5 before blocking with 0.4% Triton X-100, 10% FBS and 10% goat serum (GS) for 30 min. Incubation with primary antibody was performed in PBS containing 1% GS overnight at 4°C. The secondary antibody incubation was for 1 h at room temperature and nuclei were subsequently stained with Hoechst 33342. BrdU staining was examined with a Nikon Eclipse 55i microscope (Nikon) and images were captured with a Digital Sight DS-5 M digital camera (Nikon).

### Myogenic differentiation assay

After 5 days in proliferation medium, satellite cells were shifted to differentiation medium (IMDM supplemented with 2% horse serum, Gibco, 100 U/ml penicillin, 100 μg/ml streptomycin and 50 μg/ml gentamycin). After 48 hours cells were fixed with 4% PFA in PBS and immunostained for anti-sarcomeric myosin MF20 antibody (final dilution: 1:25; The Developmental Studies Hybridoma Bank). Nuclei were stained with Hoechst 33342. Samples were examined with a Nikon Eclipse 55i microscope (Nikon) and images were captured with a Digital Sight DS-5 M digital camera (Nikon). Fusion index was determined as the number of myosin-expressing myotubes with 1, 2, 3–4 or ≥5 nuclei versus the total number of myosin-expressing myotubes in the area analyzed.

### Statistics

Statistical analysis was performed using two-tailed Student's t-test or one-two way ANOVA when appropriate. (*, P<0.05; **, P< 0.01; ***, P< 0.001).

## Results

### T cells infiltrate muscle after acute injury

**Fig 1:**
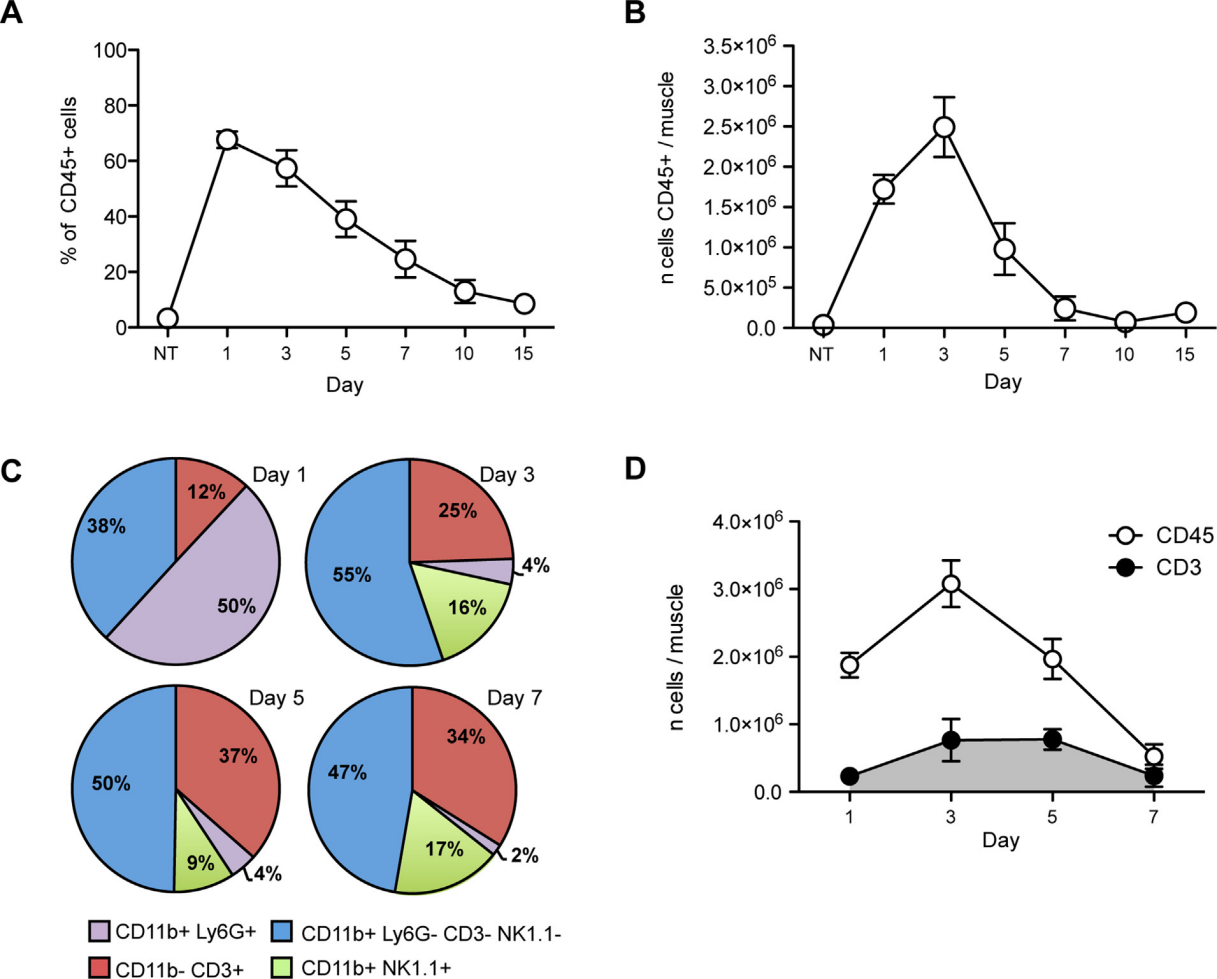
CD45^+^ cells, including T cells, infiltrate skeletal muscle after CTX injury. (A) Percentages of CD45^+^ cells in skeletal muscle at different time points after CTX injury (n ≥ 3 mice analyzed per time point, mean ± SEM). (B) The number of CD45^+^ cells in skeletal muscle peaks at day 3 and rapidly decreases thereafter (n ≥ 3 mice per time point, mean ± SEM). (C) The percentage of T cells (red) among muscle-infiltrating CD45^+^ cells increase with time, passing from a few cells present at day 1 (12 ± 3%, mean ± SD) to 25 ± 7% (mean ± SD) at day 3, and 37 ± 7% and 34 ± 7% (mean ± SD) at day 5 and 7 respectively (n ≥ 4 mice analyzed per time point). (D) The number of CD45^+^ and CD3^+^ cells present in muscle at different time points after injury. CD3^+^ cells increase in number after day 3, remain constant until day 5, and decline by day 7 (n ≥ 3 mice per time point, mean ± SEM).

To quantify the involvement of the adaptive immune system in the immediate and sustained response to skeletal muscle injury, we injected cardiotoxin (CTX) in the *tibialis anterior* (TA) and *quadriceps* muscles of young, healthy C57BL/6 mice. At various time points after this acute injury, we collected and digested the muscle and retrieved mononuclear cells, which were then characterized by flow cytometry. Relatively few CD45^+^ cells were detected in unmanipulated muscle (about 3% of the cells retrieved after enzymatic digestion, NT Fig 1A). After injury, the number of CD45^+^ leukocytes rapidly increased, peaking at day 3 (Fig 1B) and abating thereafter, as described [2, 3]. 10 days after injury CD45^+^ cells virtually disappeared (Fig 1A and 1B), coincident with muscle regeneration (S1 Fig, upper panel). Taking advantage of multicolor flow cytometry, which allows simultaneous visualization of several antigens, we identified and quantified the relative fraction of neutrophils (CD11b^+^ Ly6G^+^), CD11b^+^ cells (population that includes macrophages, eosinophils and mast cells), T cells (CD11b^-^ CD3^+^), B cells (CD11b^-^CD19^+^) and NK cells (CD11b^+^NK1.1^+^) that infiltrate the muscle at various times after injury (Fig 1C and S2 Fig). At early time points, CD45^+^ CD11b^+^ Ly6G^+^ neutrophils and CD45^+^ CD11b^+^ Ly6G^-^ CD3^-^ NK1.1^-^ cells were the most abundant infiltrating cells, accounting together for 90% (88 ± 3%, mean ± SD) of the leukocytes present (Fig 1C). Relatively few T cells (CD45^+^ CD11b^-^ CD3^+^ cells; 12 ± 3%; Fig 1C and 1E in S2 Fig) were detectable. At day 3, neutrophils were <5% (4 ± 1.5%) and CD11b^+^ Ly6G^-^ CD3^-^ NK1.1^-^ cells accounted for the majority (55 ± 6%; Fig 1C) of infiltrating cells. Starting from day 3 and continuing to day 7, the infiltrate included a progressively growing percentage of T cells (up to the 37 ± 7.4% of the muscle CD45^+^ cells at day 5, Fig 1C). The number of T cells in the muscle peaked at day 3 and remained constant through day 5 before declining at day 7 after injury (Fig 1D). Variable percentages of NK cells (CD45^+^CD11b^+^NK1.1^+^, Fig 1C and Fig 1H in S2 Fig) were observed to be present from day 3 to day 7, whereas we never observed infiltrating B cells (CD45^+^ CD11b^-^CD19^+^ cells, Fig 1C and Fig 1G in S2 Fig). Thus, T cells are specifically enriched at sites of muscle injury, and appear with delayed kinetics compared to phagocytes.

### Lymphocytes contribute to skeletal muscle regeneration

**Fig 2:**
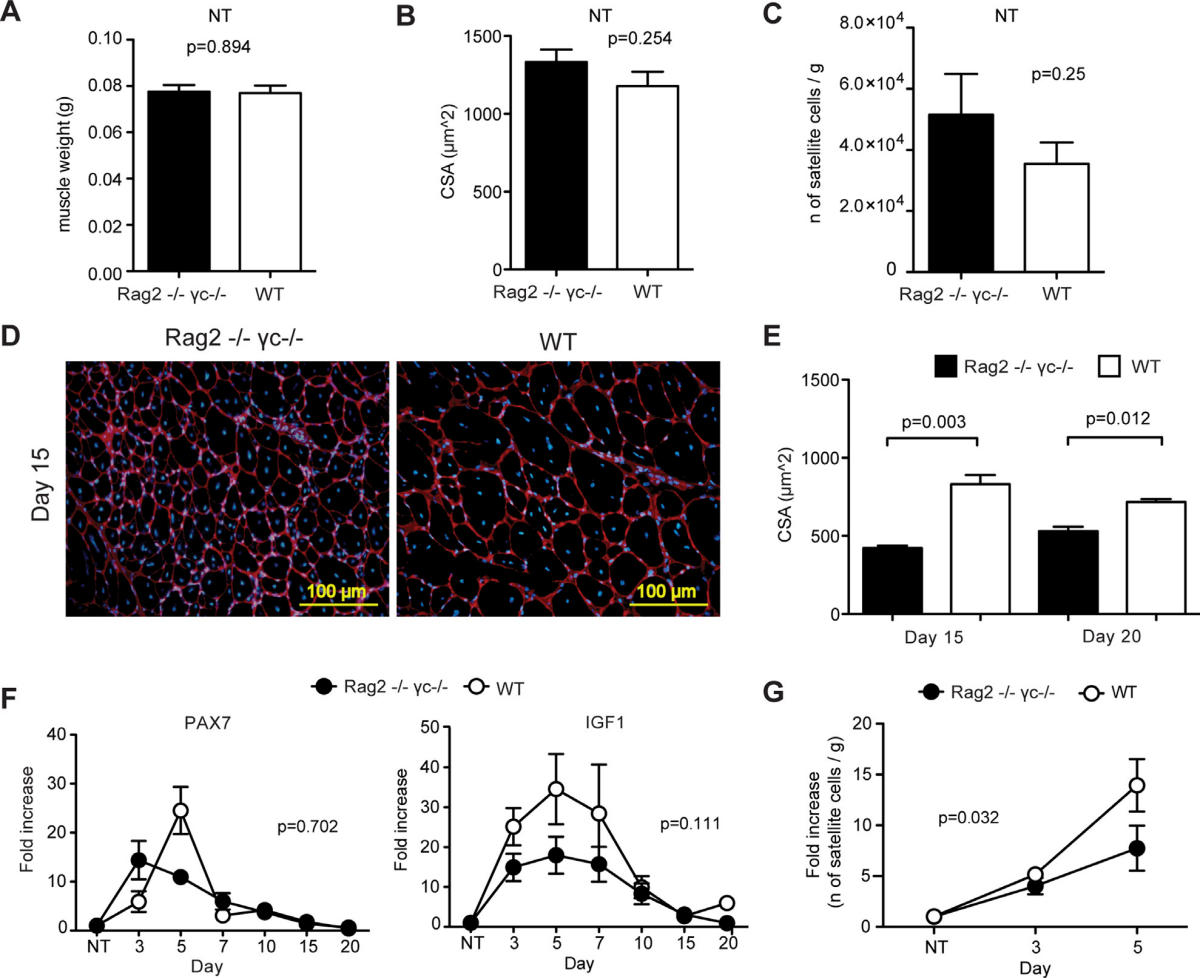
Skeletal muscle regeneration is impaired by the absence of T cells. Muscle weight (A), cross-sectional area (CSA) of myofibers (B) and the number of satellite cells (C) are comparable in uninjured Rag2^-/-^ γ-chain^-/-^ (black bars) and C57Bl6 (WT, white bars) mice (n = 4 mice per group, mean ± SEM). (D) Representative images of skeletal muscle sections at day 15 after CTX. Centrally nucleated myofibers (new formed fibers) are shown (Red = Laminin; Blue = Hoechst). (E) At day 15 and 20 after injury, the CSA of myofibers is significantly lower in the absence of T lymphocytes (Rag2^-/-^ γ-chain^-/-^ mice, black bars) (n ≥ 2 mice per group, mean ± SEM). (F) Pax7 expression peaks at day 5 is blunted in Rag2^-/-^ γ-chain^-/-^ mice (black circles); the same mice did not show differences in the level of expression of Igf1 compared to WT mice (white circles) (n ≥ 2 mice per group, mean ± SEM; fold increase was calculated with reference to the untreated muscle, NT). (G) Rag2^-/-^ γ-chain^-/-^ mice (black circles) show a blunted expansion of satellite cells at 5 days after injury compared to WT mice (white circles) (n ≥ 3 mice per group, mean ± SEM; fold increase was calculated with reference to the untreated muscle of the corresponding genotype, NT).

Because of defective V(D)J recombination and deletion of the common cytokine receptor γ-chain, Rag2^-/-^ γ-chain^-/-^ mice lack T, B, NK, gamma/delta and NKT lymphocytes [10]. These mice thus represent an useful tool to assess the role of adaptive immunity in the response to muscle injury. Analysis of uninjured skeletal muscle in Rag2^-/-^ γ-chain^-/-^ mice revealed muscle weight and fiber size (cross-sectional area, CSA) comparable to WT mice (Fig 2A and 2B). At 15 days after injury, centrally nucleated regenerating myofibers were evident in both WT and Rag2^-/-^ γ-chain^-/-^ mice (Fig 2D). At 15 and 20 days after injury, we measured the CSA of regenerating myofibers, and found that regenerating myofibers in Rag2^-/-^ γ-chain^-/-^ mice were significantly smaller than those in WT mice (Fig 2E), suggesting a defect in the myogenic process.

We next assessed by qRT-PCR in the whole muscle of Rag2^-/-^ γ-chain^-/-^ and of WT mice the expression of the satellite cells marker, *Pax7* [11] and of *Igf1*, a key regulator of protein synthesis and skeletal muscle mass during postnatal muscle development [12–14]. In the absence of lymphocytes, the peak of *Pax7* expression, visible at day 5 in WT mice, appeared to be blunted, although this difference did not reach statistically significance (p = 0.702). We could not detect significant differences in *Igf1* expression either (Fig 2F). To assess directly the number of satellite cells present in the tissue, we quantified by flow cytometry (S3A Fig) the number of skeletal muscle satellite cells (identified as CD45^-^CD11b^-^Ter119^-^Sca1^-^B1int^+^CXCR4^+^, [15]) at days 3 and 5, time points that should include the major satellite cell expansion response necessary for appropriate muscle regeneration. In untreated muscles, the number of satellite cells was comparable in the two mouse models (Fig 2C). In line with the *Pax7* expression data, we observed a reduction in the expansion of satellite cells in Rag2^-/-^ γ-chain^-/-^ mice compared to WT mice at day 5 after injury (P = 0.03, two-way ANOVA; Fig 2G). In contrast, we did not observe any significant difference in the number of fibro-adipogenic precursor cells (FAPs, CD45^-^CD11b^-^Ter119^-^Sca1^+^ [16]) or hematopoietic cells (CD45^+^CD11b^+^Ter119^+^) at the same time points after in injury between WT and Rag2^-/-^ γ-chain^-/-^ mice (S3A and S3B Fig). Together these data suggest that the absence of lymphocytes in Rag2^-/-^ γ-chain^-/-^ mice leads to an impairment of satellite cell expansion after acute injury.

### Lymphocytes that infiltrate the muscle are enriched in CD4^+^CD25^+^FOXP3^+^ T regulatory cells

**Fig 3:**
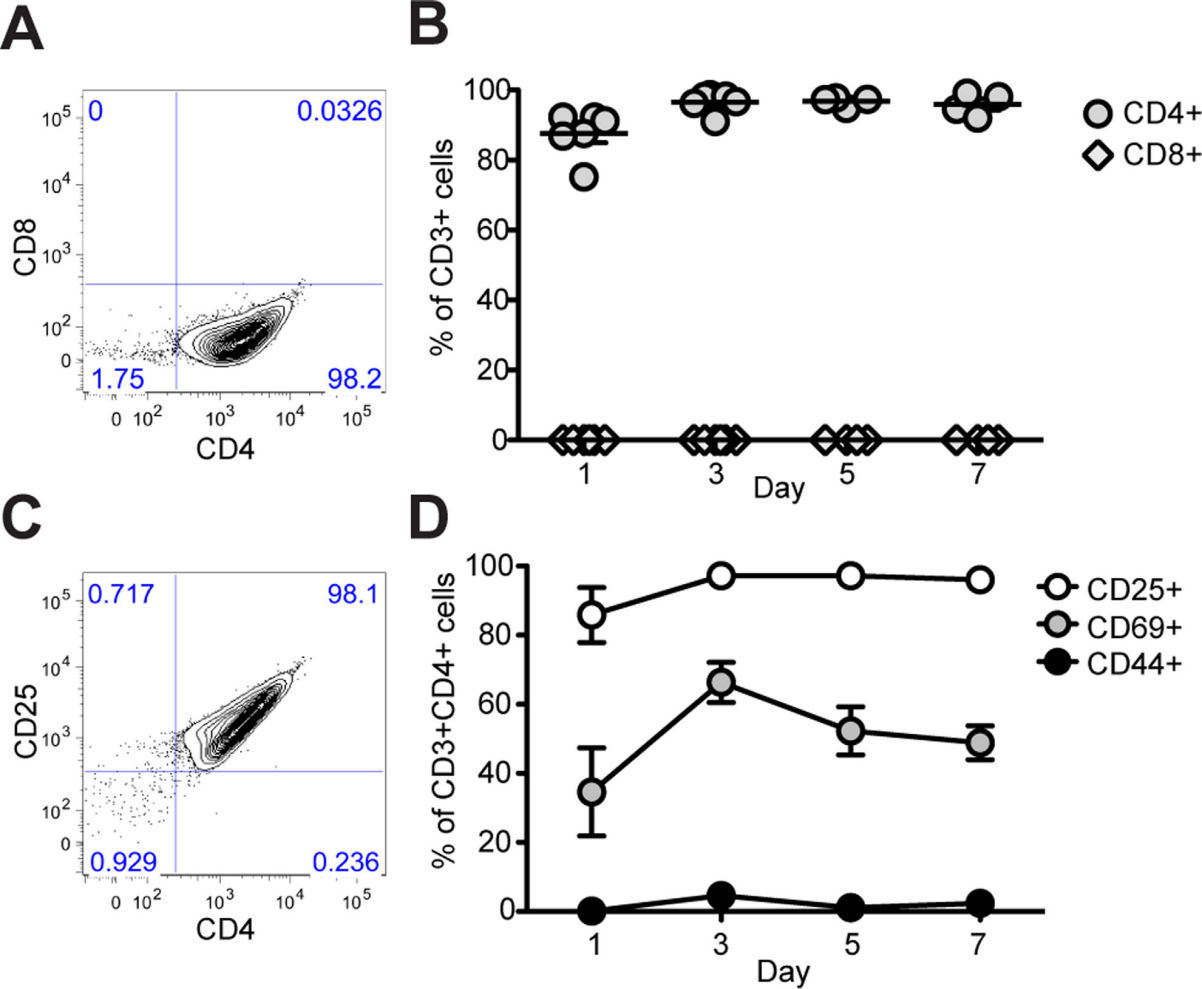
Muscle-infiltrating T cells are CD4^+^ and express CD25 and CD69. (A–B) Flow cytometric analysis of CD4 and CD8 expression on CD3^+^ T cells harvested from injured skeletal muscle. A representative plot from day 3 is shown at left and data are summarized at right as % CD4^+^ or % CD8^+^ at each time point analyzed (n ≥ 4 mice per time point, mean ± SEM). (C–D) The majority of CD3^+^CD4^+^ cells express CD25 (range: 86–97%), CD69 (34–66%), but not CD44 (0.1–4%) (n ≥ 4 mice per time point, mean ± SEM).

Our data in Rag2^-/-^ γ-chain^-/-^ mice indicate that the adaptive immune system, although not absolutely required, contributes significantly to the proper regeneration of skeletal muscle. In order to better understand which population(s) of T cells may be involved in regulating muscle repair, we characterized by flow cytometry the lymphocytes that infiltrate the injured muscle of WT mice and observed that the infiltrating cells consistently express CD4 and never, at any analyzed time point, expressed the CD8 marker (Fig 3A and 3B). At the same time points, we analyzed the expression of CD25, CD69 and CD44 in the CD3^+^ CD4^+^ gate (Fig 3C and 3D and S4 Fig). The majority of CD3^+^CD4^+^ cells expressed the IL2 receptor, CD25 (86 to 97%), and the early activation marker, CD69 (34 to 66%), but lacked the cell adhesion molecule, CD44 (0.1 to 4%) (Fig 3C and 3D and S4 Fig). Thus, activated T helper cells appear to be selectively recruited or expanded within injured skeletal muscles.

**Fig 4:**
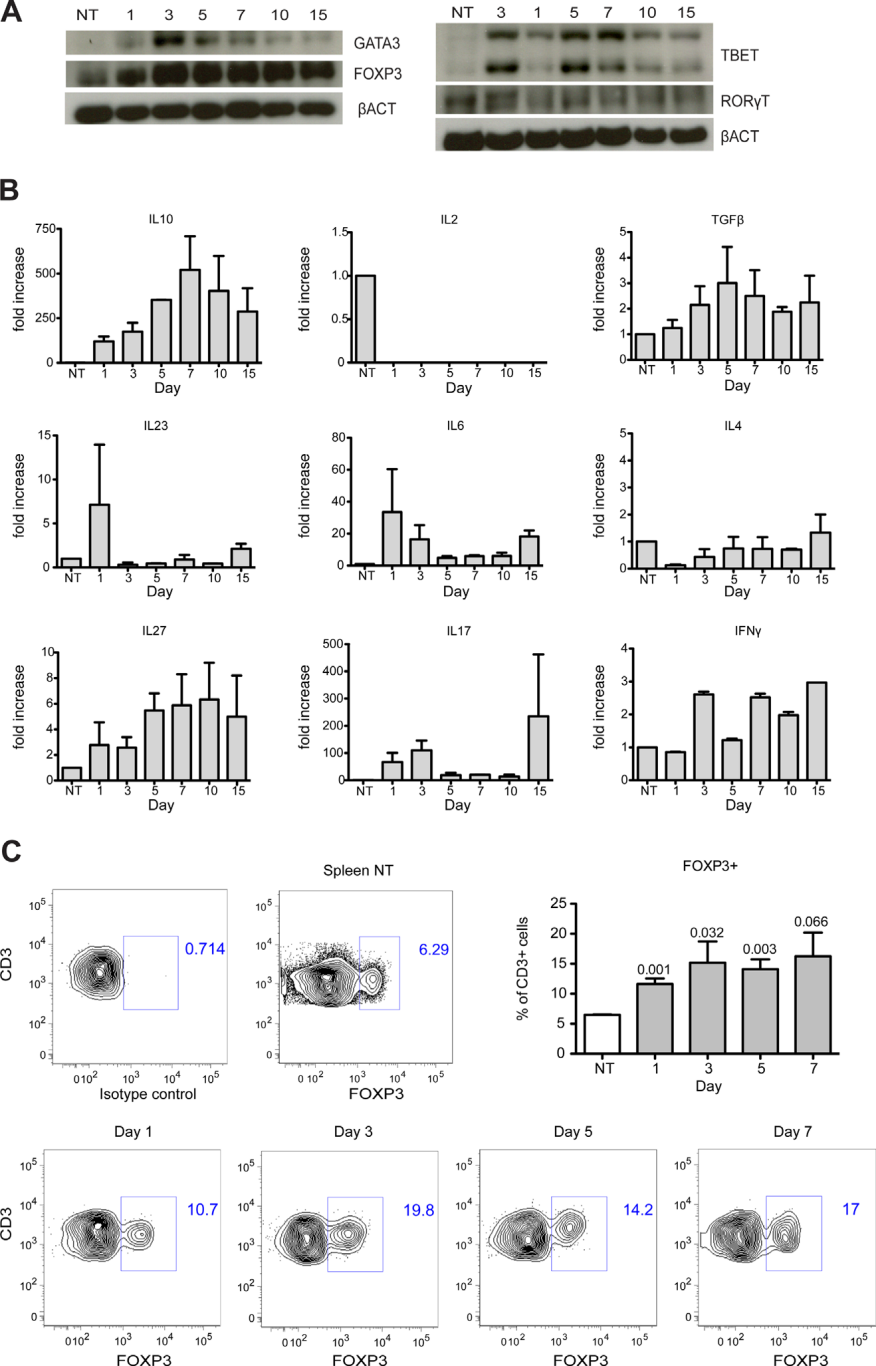
Infiltrating T cells express FOXP3 and IL10. (A) Western Blot analysis of CD3^+^ cells retrieved from skeletal muscles at different time points after muscle injury (representative blots of two experiments are shown). Muscle T cells express FOXP3 at all time points analyzed. (B) T cells isolated from skeletal muscle after CTX injury express high level of Il10. CD3^+^ cells from muscle did not express Il2, Il4 or Il23 and showed variable expression of Tgfβ, Il6, Il27, Ifnγ and Il17 (n = 2 independent experiments, mean ± SEM). (C) Flow cytometric analysis of FOXP3 expression on CD3^+^ T cells. Representative plots of each time point analyzed are shown. The percentage of CD3^+^FOXP3^+^ cells in the injured muscle were quantified and compared to untreated spleens (n ≥ 3 mice per time point, mean ± SEM; t-test) (NT = CD3^+^ cells from untreated mouse spleen).

T helper cells comprise at least four lineages: Th1, Th2, T regulatory (Treg) and Th17 [17, 18], each characterized by the expression of specific transcription factors (TBET, GATA3, FOXP3 and RORγt, respectively) and by the production of particular cytokines [17]. 1, 3, 5, 7, 10 and 15 days after damage, we retrieved CD3^+^ cells by immuno-magnetic sorting. Western blot analysis for TBET, GATA3, FOXP3 and RORγt on the lysates of CD3^+^ cells revealed robust expression of FOXP3, maintained throughout muscle regeneration (Fig 4A). Also GATA3, TBET and RORγT, were found to be expressed by infiltrating CD3^+^ cells. However, their expression peaked between day 3 and day 5, and decreased by day 10, a time at which FOXP3 was still robustly expressed (Fig 4A). qRT-PCR analysis of selected cytokines revealed that CD3^+^ cells abundantly expressed *Il10* and some *Tgfβ*. In contrast, *Il2* was undetectable and *Il4*, *Il6*, *Il27*, *Il23*, *Ifnγ* and *Il17* showed inconsistent and less robust expression (Fig 4B). Sustained expression of FOXP3 is central to the identity of the Treg lineage, which restrains the activation and responses of both innate and adaptive immune cells [19]. Other features of muscle lymphocytes, including expression of CD25 and production of IL10 and TGFβ but not of IL2 [20] are as well rather characteristic features of Treg [21]. Flow cytometry analysis confirmed that a consistent fraction of CD3^+^ cells expressed FOXP3, thus confirming the presence of Treg cells at day 1, 3, 5, and 7 after injury (Fig 4C).

### T regulatory cells induce skeletal muscle precursor cell expansion

**Fig 5:**
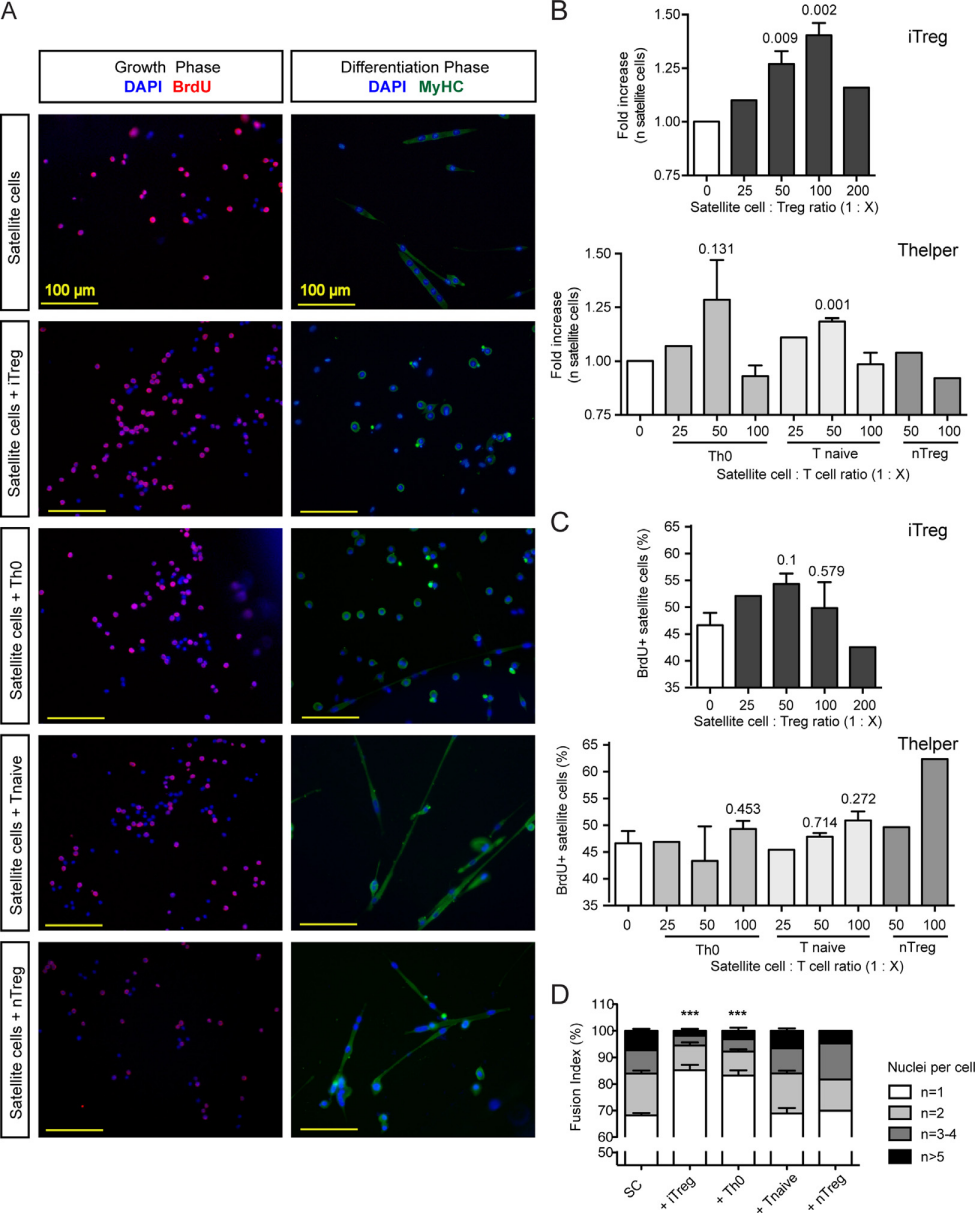
iTreg cells induce satellite cell expansion in vitro, but inhibit their differentiation. (A) Satellite cells were cultured in a myogenesis assay in the presence of iTreg, Th0, Tnaive and nTreg cells and evaluated for their ability to proliferate in the growth phase and to fuse to form myotubes in the differentiation phase (Red = BrdU, Green = MYOSIN HEAVY CHAIN (MyHC), Blue = DAPI, DNA). (B) After 3 days the number of satellite cells was significantly higher in the presence of iTregs (at the 1:50 and 1:100 ratios, n = 3 independent experiments, mean ± SEM, fold increase was calculated with reference to the satellite cell alone condition). (C) After 72 hours of co-culture, satellite cells were pulsed with BrdU for 2 hours and the percentage of BrdU^+^ satellite cells was calculated (n = 3 independent experiments, mean ± SEM). (D). Satellite cells produced fewer elongated, multinucleated myotubes when induced to differentiate in the presence of iTregs. The fusion index (n of nuclei per cell) was calculated for satellite cells differentiated in presence of iTreg, Th0, Tnaive or nTreg. Both iTreg and Th0 cells inhibited satellite cell differentiation (1:50 ratio for all the conditions, n = 3 independent experiments, mean ± SEM; n = number of nuclei per cell).

Given the essential role of satellite cells in muscle regeneration [22–24], we wondered whether Treg cells might directly influence the ability of satellite cells to proliferate and/or differentiate. To address this question, we sorted satellite cells by FACS from skeletal muscles of Pax7-zsgreen mice and carried out myogenic differentiation assays in the presence of *in vitro* derived Treg cells (iTreg), natural Treg (nTreg), naive CD4^+^ T cells or activated non-polarized CD4^+^ cells (Th0). We followed satellite cell fate during two phases of the myogenesis assay—the growth phase, during which satellite cells proliferate, and the differentiation phase, during which they elongate, fuse and generate multinucleated myotubes (Fig 5A). We observed that the number of satellite cells was significantly higher in presence of iTreg (1:50–1:100, Fig 5B). To determine if the increased number of satellite cell reflected an increased proliferation rate, we performed a BrdU incorporation assay in which the number of BrdU^+^ cells was quantified after a 2 hour pulse of BrdU. The percentage of BrdU^+^ cells was slightly, but not significantly, increased in presence of iTreg (Fig 5C). Longer interaction with iTregs resulted in a lower number of satellite cells that elongated and fused to form myotubes (Fig 5A and 5D), indicating that the interaction between satellite cells and Tregs sustains early satellite cell expansion, but can delay their terminal myogenic differentiation. None of the other T cell populations assayed had any effect on satellite cell proliferation or differentiation, with the exception of Th0 cells, which showed a mild effect on satellite cells expansion at a 1:50 ratio and inhibited the differentiation of myogenic precursors (Fig 5). Flow cytometry analysis indicated that satellite cells express negligible surface levels of IL10 receptor (S3C and S3D Fig) suggesting that FOXP3^+^ cells influence satellite cell functions in an IL10 independent manner.

## Discussion

Acute skeletal muscle injury is followed by a stereotypic inflammation during which first neutrophils and then macrophages invade the tissue. Macrophages are responsible for the removal of cellular debris produced by muscle injury and create an environment that is permissive to satellite cell proliferation and differentiation [3]. In contrast to the clear role of the innate immune system in skeletal muscle regeneration, less is known regarding the involvement of the adaptive immune system. The presence of T lymphocytes in the context of chronic muscle damage [6, 7] and the fact the muscle fibers are able to function as non-professional antigen presenting cells [25] suggest that neutrophils and macrophages may not be the only immune cells involved in muscle regeneration.

We characterized by flow cytometry the immune infiltrate at the site of muscle injury at different time points after CTX damage and confirmed the kinetics of cell infiltration described previously in literature [3]: at 24 hours after the damage the majority of immune cells were neutrophils, which then rapidly disappeared. After 3 days, the majority of cells (around 60%, Fig 1) were CD11b+ cells. At the same time, CD3^+^ T cells increased in number at the site of injury, peaking in number at 3 days after injury and remaining at these higher numbers until day 5 (Fig 1). Virtually no B-lymphocytes and few NK cells are present during muscle regeneration. Some recent papers show that NK cells participate in tissue regeneration in other contexts [26, 27]. Future studies should examine their possible involvement in skeletal muscle regeneration. Compared to other studies (i.e. [28]) our characterization shows a different extent of lymphocyte infiltration of the skeletal muscle. This discrepancy could be explained by the different experimental procedures used to liberate infiltrating leukocytes from the tissue, and specifically by the enzymes used to digest the muscle, and to analyze retrieved cells. Moreover, differences in mouse strain and housing facilities could be involved [29].

The period during which T cells are particularly abundant in the muscle (3 to 5 days after injury), is a crucial time during which macrophages are known to switch from a pro-inflammatory M1 phenotype to an anti-inflammatory M2 phenotype [2]. This time point is also coincident with the peak of satellite cell proliferation [16, 30]. To understand if T cells are mere bystanders to these events or participate in regulating the regeneration process, we applied the CTX injury model on Rag2^-/-^ γ-chain^-/-^ mice. Rag2^-/-^ γ-chain^-/-^ mice are deficient in multiple lymphocyte cell types, but retain functional macrophages and neutrophils [31]. We observed that muscle is able to regenerate in the absence of T cells, as at 15–20 days after injury we saw centrally nucleated myofibers and no signs of necrosis (Fig 2D and S1 Fig, lower panels); however, the regenerated myofibers in Rag2^-/-^ γ-chain^-/-^ mice were significantly smaller than those in wild type mice (Fig 2D and 2E). We did not observe significant differences in the expression of myogenic molecules like *Pax7* and *Igf1* in Rag2^-/-^ γ-chain^-/-^ muscle compared to wild type (Fig 2F). These results are consistent with those of Lu et al, who found that macrophages serve as the predominant source of IGF1 during muscle regeneration [32]. We did observe an apparent alteration in the kinetics of *Pax7* expression during regeneration in Rag2^-/-^ γ-chain^-/-^ mice, particularly between days 3 and 5, suggesting an impact on satellite cell proliferation. To test this hypothesis, we quantified by flow cytometry the number of satellite cells at days 3 and 5 and showed that Rag2^-/-^ γ-chain^-/-^ mice exhibit a slight, but significant decrease in satellite cell number compared to WT at day 5 (Fig 2G), consistent with a role for adaptive immune cells in the proliferation of muscle satellite cells.

To identify the lymphocytes that might contribute to the regulation of muscle regeneration, we next characterized the muscle infiltrating T cells and observed that only CD4^+^ T helper cells were detectable in regenerating muscle (Fig 3B), making it unlikely that CD8^+^ cytotoxic lymphocyte were involved in muscle damage or repair after acute injury. Activated T helper cells, expressing CD25 and CD69 (Fig 3D), infiltrated skeletal muscle after acute injury and showed characteristics of T regulatory cells, including expression of FOXP3 at all the time points analyzed (Fig 4A and 4C), and expression at the mRNA level of high levels of *Il10*, some *Tgfβ*, but no *Il2* (Fig 4B) [33–35]. The expression of other cytokines analyzed (*Il4*, *Il6*, *Il27*, *Il23*, *Ifnγ and Il17*) was variable and inconsistent, in line with expression of the related transcription factors (Fig 4A and 4B). In summary, we show that acute sterile injury results in a selective recruitment of T cells in skeletal muscle and that these T cells are enriched for CD4^+^ FOXP3^+^ T regulatory cells. Further studies are required to identify the signals involved in Treg recruitment and expansion at sites of muscle damage. Acute muscle injury is associated with the local generation of DAMPs (Damage-Associated Molecular Pattern Molecules) such as HMGB1 (High-Mobility Group Box 1) [9, 36], which are well known to possess chemotactic activity [37] and to regulate the functions of T cell subsets [38]. However, several other pathways, including those associated with activation of the complement system [39] could also be involved.

An actual regulatory function of Treg on other T cells appears unlikely in the system we have explored in this study. Such action could be crucial, however, in other persistent pathological states of skeletal muscle, such as DMD and polymyositis/dermatomyositis. In these chronic muscle diseases, CD8^+^ T cells are present and activated, and failure to regulate these cells might be associated with eventual tissue wasting [40, 41]. In contrast, in the normal regenerative response to muscle injury, Treg could accomplish their function by directly interacting with muscle cells, by interacting with macrophages, or both. A population of Tregs was recently suggested to regulate muscle regeneration in part via modulating the switching of M1 to M2 macrophages and by the secretion of Amphiregulin, which in turn could influence the myogenicity of satellite cells [42].

Our data indicate that the Tregs in injured muscles indeed prompt the expansion of resident muscle stem cells. This may be a common strategy in tissue repair, as T cells contribute to regenerative responses in the liver after partial hepatectomy [43] and regulate the efficacy of bone healing [44]. In the bone healing system, IL10 expression and upregulation of angiogenic factors appear essential [44], while persistence of terminally differentiated CD8^+^ effector memory T cells results in delayed or incomplete fracture healing [45]. To explore the possibility that Tregs directly interact with muscle satellite cells, we performed co-culture experiments in which we monitored both the proliferation and differentiation capacity of satellite cells (Fig 5A). We observed a significant increase in satellite cell numbers in the presence of *in vitro* induced Tregs (iTreg, Fig 5B). BrdU incorporation assays showed a slight increase in the percentage of BrdU^+^ cells in the same condition, suggesting that iTregs likely induce expansion of satellite cells *in vitro* by promoting both proliferation and survival of the cells. This result fits well with our *in vivo* observations, as we see that the greatest presence of T cells in injured muscle occurs during the satellite cell proliferation phase (from days 3 to 5 after injury, Fig 1D). In addition, absence of T cells reduces the expansion of satellite cells (Fig 2G). As we observed that satellite cells express relatively low levels of IL10 receptor at the cell surface (S3C and S3D Fig), molecules other than IL10 are likely to be involved in the Treg-induced expansion of satellite cells.

The presence of Treg in culture also influences satellite cell differentiation. We observed that if we maintained Treg in culture during the differentiation phase, satellite cells were delayed in elongation and fusion to form myotubes (Fig 5C and 5D). It is possible that timing is a critical factor in the interaction of Tregs and satellite cells, and that the persistent presence of Tregs (as in our culture assays) is detrimental for skeletal muscle differentiation. *In vivo*, the interaction between Treg and muscle cells is limited in time as the number of infiltrating T cells declines after 5 days (Fig 1D). Persistent contact between T and muscle cells therefore is unlikely to occur physiologically during the acute response to injury. However, persistence of muscle-infiltrating T cells is a hallmark of DMD and inflammatory myopathies, and further studies are needed to understand the possible implications of our data in the context of chronic injury. In this context, in which different immune cells persist in inflamed tissues, Tregs could play both a detrimental and a beneficial role by influencing the fibrotic outcome of the regenerative process and the activation of stem cells. A recently published study reports that Tregs number is increased in DMD patients and in mdx mice and suggests that Tregs control inflammation by restraining IFNγ production with beneficial effects for muscle regeneration [46].

In the *in vitro* co-culture experiment we also observed a slight effect of un-polarized, activated Th0 cells on myogenic differentiation. Further studies will be needed to determine the effect of proliferating muscle precursor cells on T cells. In the same *in vitro* system, non-activated natural Treg cells (nTregs, Fig 5) did not show any effect on satellite cells. It is possible that activation of T cells is required for exerting their effects on satellite cell proliferation, although further experiments are needed to verify this hypothesis. Nonetheless, this notion is further supported by a recent publication showing that activated T cells secrete myogenic molecules that promote the proliferation and inhibit the differentiation of the immortalized myoblast cell line C2C12 [47].

In summary, our study shows that Tregs in injured skeletal muscles regulate the expansion of satellite cells, influencing the outcome of the regenerative process. Future studies unfolding the mechanisms underlying the regulation of muscle regeneration by Tregs will likely reveal new targets for promoting rapid and complete recovery from muscle damage.

## Supporting Information

S1 Fig Skeletal muscle regeneration after injury in WT and Rag2^-/-^ γ-chain^-/-^ mice. Representative hematoxylin and eosin staining of injured TA muscles from WT (upper panel) and Rag2^-/-^ γ-chain^-/-^ mice (lower panel) at 5 (left panel) and 10 days (right panel) after injury.
(TIF)

S2 Fig Representative FACS plots of muscle infiltrating leukocytes, gating strategy. (Figure A) Cells were selected based on physical parameters: doublets and debris were excluded. (Figure B) Hoechst positive and (Figure C) Live Dead negative cells were then selected and finally (Figure D) CD45^+^ cells were analyzed for the expression of (Figure E) CD11b and CD3; (Figure F) Ly6G (Figure G) CD19 and (Figure H) NK1.1. (Representative plots from muscle at 5 days after injury analysis are shown, except for Ly6G for which Day 1 and Day 7 are shown. FMO = fluorescence minus one, indicating the absence of one particular antibody in the staining).
(TIF)

S3 Fig Satellite cells *in vivo* quantification and IL10R expression analysis. (Figure A) Gate strategy for the flow cytometric quantification of satellite cells (PI - CA+ CD45-Mac1-Ter119-Sca1-B1int+CXCR4+, RED boxes), Fibro-adipogenic precursor cells (FAPs, PI - CA+ CD45-Mac1-Ter119-Sca1+, BLUE box) and hematopoietic cells (PI - CA+ CD45+Mac1+Ter119+, GREEN box). (Figure B) Quantification of FAPs and hematopoietic cells in Rag2^-/-^ γ-chain^-/-^ (black) and WT (white) mice in not injured (NT) and injured muscles at day 3 and 5 after CTX injection shows that there is no difference in these populations after injury. (Figure C) Satellite cells (PI - CA+ CD45-Mac1-Ter119-Sca1- B1int+CXCR4+, RED boxes figure A) and hematopoietic cells (PI - CA+CD45+Mac1+Ter119+, GREEN box figure A) expression of IL10Ra by flow cytometry. Satellite cells do not express IL10 receptor before (NT, blue line) or after CTX injury (Day 3, red line). (Figure D) Relative fluorescence intensity (RFI, calculated as the ratio of the Mean Fluorescence Intensity of the sample divided by Mean Fluorescence Intensity of the isotype control) of IL10Ra. Hematopoietic cells show robust expression of IL10Ra both in the untreated (NT, 2.7 ± 0.2 mean ± SD, n = 3) and in the injured muscle (Day 3, 2.5 ± 0.4 mean ± SD, n = 3). Satellite cells show very low expression of IL10Ra in the untreated (NT, 1.4 ± 0.1 mean ± SD, n = 3) and in the injured muscle (Day 3, 0.7 ± 0.2 mean ± SD, n = 3).
(TIF)

S4 Fig Representative FACS plots showing the expression of CD25, CD69 and CD44 in the CD45+CD3+CD4+ gate at different time points after injury. (TIF)
