# Correction: FOXP3+ T Cells Recruited to Sites of Sterile Skeletal Muscle Injury Regulate the Fate of Satellite Cells and Guide Effective Tissue Regeneration

**DOI:** 10.1371/journal.pone.0133101

**Published:** 2015-07-14

**Authors:** The PLOS ONE Staff

There are errors in the Author Contributions. The correct contributions are: Conceived and designed the experiments: AC AJW A. Mondino AAM PRQ. Performed the experiments: AC GC ER VB MV A. Monno. Analyzed the data: AC GF ER AJW A. Mondino AAM PRQ. Contributed reagents/materials/analysis tools: AJW A. Mondino AAM PRQ. Wrote the paper: AC GF AEA A. Mondino AJW AAM PRQ.

The following information is missing from the Funding section: AIRC—Associazione Italiana per la Ricerca sul Cancro (IG 11970/2011 to A.M.).

The publisher apologizes for these errors.
