# Supplementary figures and images for: FOXP3+ T Cells Recruited to Sites of Sterile Skeletal Muscle Injury Regulate the Fate of Satellite Cells and Guide Effective Tissue Regeneration
Source: PLoS One. 2015 Jun 3;10(6):e0128094. doi: 10.1371/journal.pone.0128094 (PMC4454513; doi:10.1371/journal.pone.0128094)

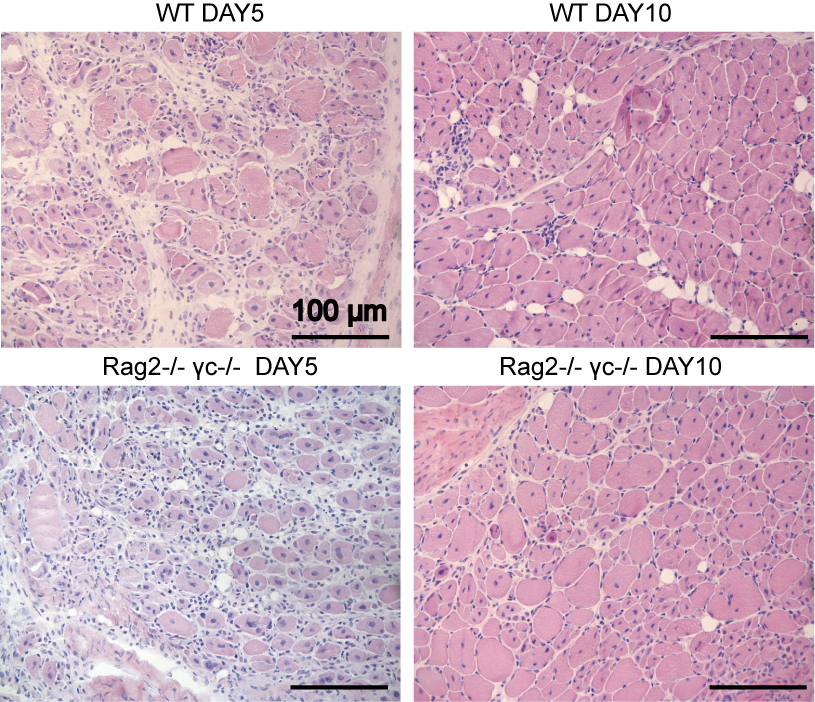

Supplement: S1 Fig — Representative hematoxylin and eosin staining of injured TA muscles from WT (upper panel) and Rag2-/- γ-chain-/- mice (lower panel) at 5 (left panel) and 10 days (right panel) after injury. (TIF) [file pone.0128094.s001.tif]

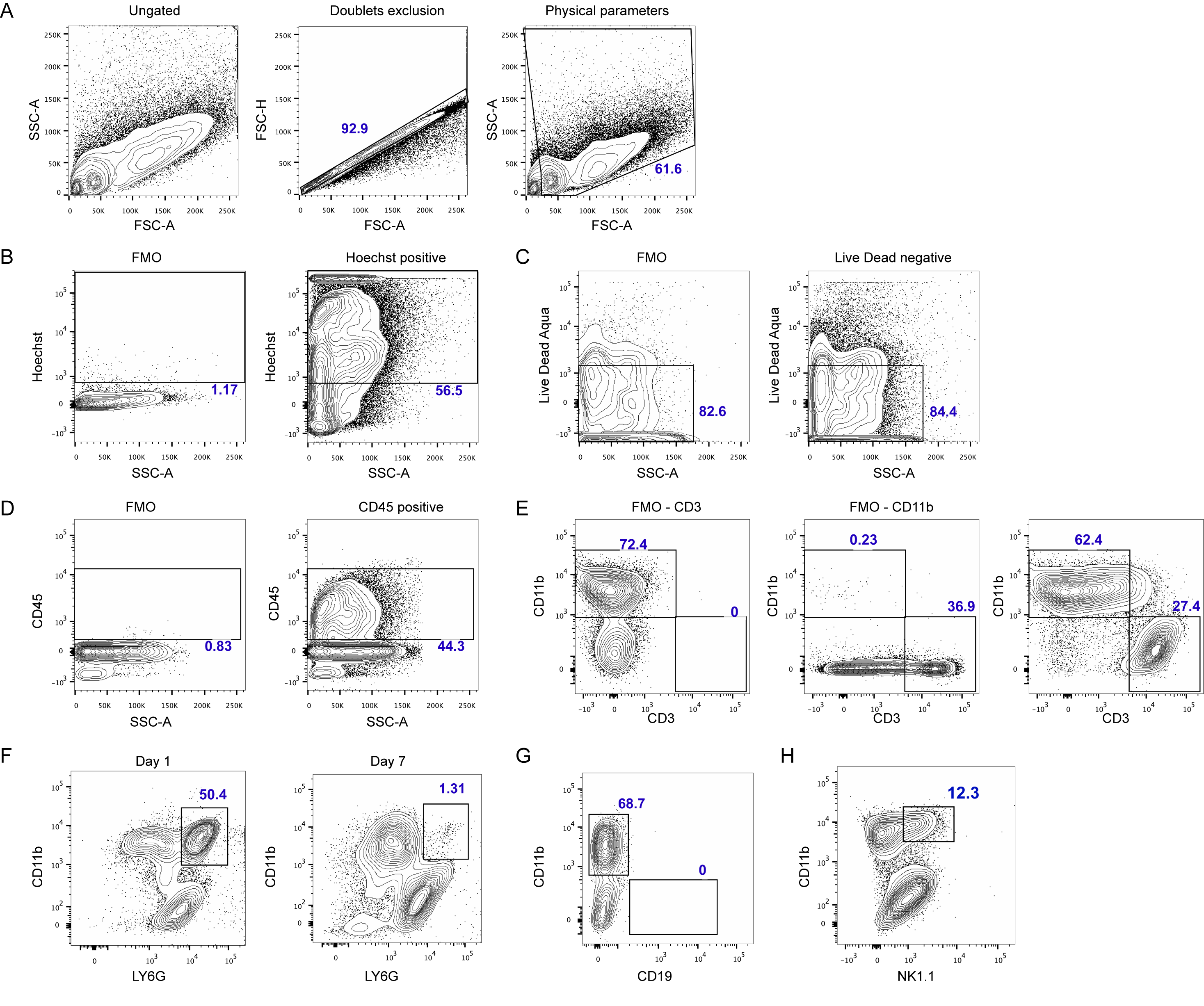

Supplement: S2 Fig — (Figure A) Cells were selected based on physical parameters: doublets and debris were excluded. (Figure B) Hoechst positive and (Figure C) Live Dead negative cells were then selected and finally (Figure D) CD45+ cells were analyzed for the expression of (Figure E) CD11b and CD3; (Figure F) Ly6G (Figure G) CD19 and (Figure H) NK1.1. (Representative plots from muscle at 5 days after injury analysis are shown, except for Ly6G for which Day 1 and Day 7 are shown. FMO = fluorescence minus one, indicating the absence of one particular antibody in the staining). (TIF) [file pone.0128094.s002.tif]

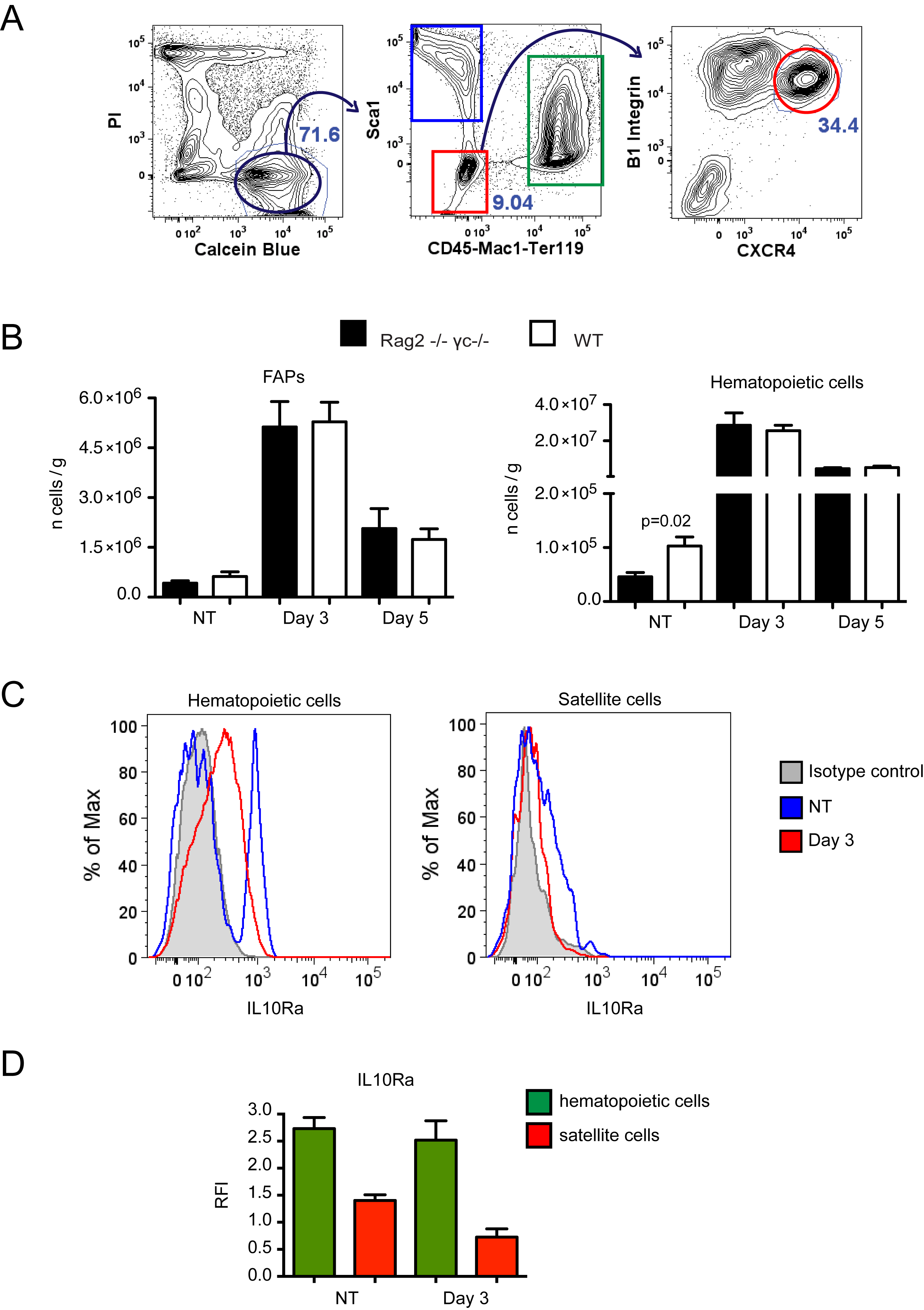

Supplement: S3 Fig — (Figure A) Gate strategy for the flow cytometric quantification of satellite cells (PI - CA+ CD45-Mac1-Ter119-Sca1-B1int+CXCR4+, RED boxes), Fibro-adipogenic precursor cells (FAPs, PI - CA+ CD45-Mac1-Ter119-Sca1+, BLUE box) and hematopoietic cells (PI - CA+ CD45+Mac1+Ter119+, GREEN box). (Figure B) Quantification of FAPs and hematopoietic cells in Rag2-/- γ-chain-/- (black) and WT (white) mice in not injured (NT) and injured muscles at day 3 and 5 after CTX injection shows that there is no difference in these populations after injury. (Figure C) Satellite cells (PI - CA+ CD45-Mac1-Ter119-Sca1- B1int+CXCR4+, RED boxes figure A) and hematopoietic cells (PI - CA+CD45+Mac1+Ter119+, GREEN box figure A) expression of IL10Ra by flow cytometry. Satellite cells do not express IL10 receptor before (NT, blue line) or after CTX injury (Day 3, red line). (Figure D) Relative fluorescence intensity (RFI, calculated as the ratio of the Mean Fluorescence Intensity of the sample divided by Mean Fluorescence Intensity of the isotype control) of IL10Ra. Hematopoietic cells show robust expression of IL10Ra both in the untreated (NT, 2.7 ± 0.2 mean ± SD, n = 3) and in the injured muscle (Day 3, 2.5 ± 0.4 mean ± SD, n = 3). Satellite cells show very low expression of IL10Ra in the untreated (NT, 1.4 ± 0.1 mean ± SD, n = 3) and in the injured muscle (Day 3, 0.7 ± 0.2 mean ± SD, n = 3). (TIF) [file pone.0128094.s003.tif]

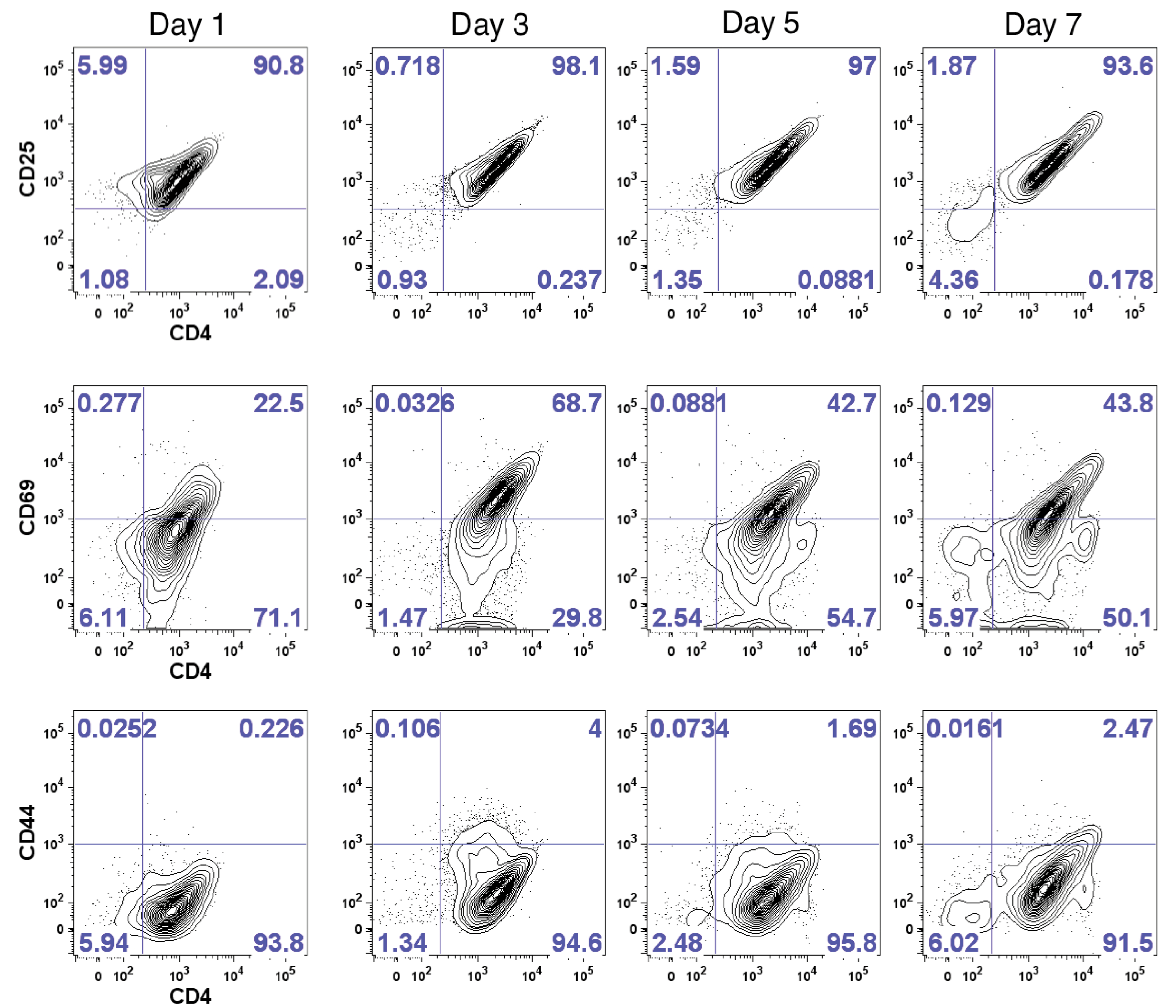

Supplement: S4 Fig — (TIF) [file pone.0128094.s004.tif]
